# Care-seeking patterns for fatal malaria in Tanzania

**DOI:** 10.1186/1475-2875-3-27

**Published:** 2004-07-28

**Authors:** Don de Savigny, Charles Mayombana, Eleuther Mwageni, Honorati Masanja, Abdulatif Minhaj, Yahya Mkilindi, Conrad Mbuya, Harun Kasale, Graham Reid

**Affiliations:** 1 Tanzania Essential Health Interventions Project, P.O. Box 78487, Dar es Salaam, Tanzania; 2 International Development Research Centre, Box 8500, Ottawa, Canada; 3 Ifakara Health Research and Development Centre, Box 56, Ifakara, Tanzania; 4 Rufiji Demographic Surveillance System, Ikwiriri, Tanzania; 5 Ministry of Health, Box 9083, Dar es Salaam, Tanzania

## Abstract

**Background:**

Once malaria occurs, deaths can be prevented by prompt treatment with relatively affordable and efficacious drugs. Yet this goal is elusive in Africa. The paradox of a continuing but easily preventable cause of high mortality raises important questions for policy makers concerning care-seeking and access to health systems. Although patterns of care-seeking during uncomplicated malaria episodes are well known, studies in cases of fatal malaria are rare. Care-seeking behaviours may differ between these groups.

**Methods:**

This study documents care-seeking events in 320 children less than five years of age with fatal malaria seen between 1999 and 2001 during over 240,000 person-years of follow-up in a stable perennial malaria transmission setting in southern Tanzania. Accounts of care-seeking recorded in verbal autopsy histories were analysed to determine providers attended and the sequence of choices made as the patients' condition deteriorated.

**Results:**

As first resort to care, 78.7% of malaria-attributable deaths used modern biomedical care in the form of antimalarial pharmaceuticals from shops or government or non-governmental heath facilities, 9.4% used initial traditional care at home or from traditional practitioners and 11.9% sought no care of any kind. There were no differences in patterns of choice by sex of the child, sex of the head of the household, socioeconomic status of the household or presence or absence of convulsions. In malaria deaths of all ages who sought care more than once, modern care was included in the first or second resort to care in 90.0% and 99.4% with and without convulsions respectively.

**Conclusions:**

In this study of fatal malaria in southern Tanzania, biomedical care is the preferred choice of an overwhelming majority of suspected malaria cases, even those complicated by convulsions. Traditional care is no longer a significant delaying factor. To reduce mortality further will require greater emphasis on recognizing danger signs at home, prompter care-seeking, improved quality of care at health facilities and better adherence to treatment.

## Background

Malaria continues to be the largest single component of the burden of disease in sub-Saharan Africa, even though simple, effective and affordable treatments exist. Malaria's pervasive morbidity and high mortality persist because of failed transactions between those at risk of malaria transmission and available preventive and curative health systems. The consequence is not just an intolerable burden for individuals, their families and national health systems, but is also a devastating and continuing impediment to socio-economic development on the continent. Unlike HIV and TB, the other major fatal communicable diseases in Africa, malaria deaths can be prevented by prompt treatment with relatively affordable and efficacious drugs. Yet this goal continues to be elusive. The paradox of a continuing, but easily preventable, cause of high mortality raises important questions for policy makers and health systems in Africa.

### Malaria in Tanzania

**Figure 1:**
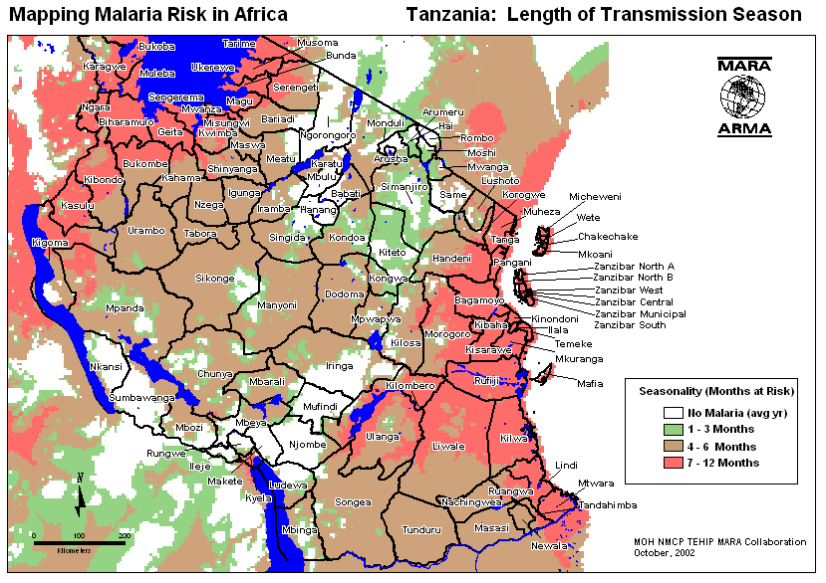
**Risk of malaria transmission. **Length of malaria transmission season in Tanzania based on the MARA climate model. (Source, Ministry of Health TEHIP and MARA-Tanzania).

The United Republic of Tanzania has a population of 34.5 million, all of whom are at risk of malaria. However, endemicity and risk of transmission varies and have recently been mapped by the MARA collaboration [1](Figure 1). This GIS-based analysis reveals that 75% of the population is subject to stable perennial or stable seasonal malaria transmission; 8% to unstable highly seasonal transmission; and 17% to no malaria transmission in the average year, but still at risk of epidemic malaria. Tanzania has the third largest population at risk of stable malaria in Africa after Nigeria and the Democratic Republic of Congo (MARA-Lite Software 3.0.0, ). Malaria is the leading cause of out-patient and in-patient health service attendance for all the ages and the leading cause of death in both children and adults in all regions of Tanzania [2]. In Tanzania, malaria is believed to be directly or indirectly responsible for about 16 million annual malaria episodes and 100,000 to 125,000 annual deaths (70–80,000 in under-fives) [3].

### National Responses

Increasing global political commitment to malaria control in recent years stimulated by the Roll Back Malaria partnership and the Global Fund to fight AIDS, TB and Malaria, has been reflected in renewed attention to malaria in Tanzanian national level policies, and to a lesser extent, in local government practices. The National Malaria Control Program's strategic plan is built around four pillars: 1) improved malaria case management; 2) national scale use of insecticide treated nets (ITNs); 3) prevention of malaria in pregnancy; and 4) malaria epidemic prevention and control [3]. Integrated Management of Childhood Illnesses (IMCI), intermittent presumptive treatment in pregnancy (IPT) and Insecticide Treated Nets (ITNS) are all part of Tanzania's national package of essential health interventions. In late 2001 the national antimalarial drug policy ceased chloroquine as the first line drug due to high drug resistance. On average there was 52% total treatment failure in sentinel surveillance of antimalarial drug efficacy [4]. The new policy includes sulfadoxine-pyrimethamine (SP) as first line, amodiaquine as second line and quinine as third line antimalarials. In 1998 a district-scale, and later in 2000, a national-scale social marketing programme for ITNs was implemented by the Ministry of Health and its NGO and donor partners in order to develop and test processes for increasing affordable supply, demand and coverage for ITNs and to stimulate the commercial market for ITNs. As part of the health sector reforms, a sector-wide approach to financing places per capita resources under the control of local government councils at district level where they can be used to support the provision of the national package of health interventions, including malaria interventions at both public and non-governmental health facilities.

### Household responses

Tanzanians enjoy relatively good geographic access to primary health services, with 90% of the population within one hour of a government health service [5]. Government health services for children under five years of age and for pregnant women are officially free. However, household health needs and demands are great. Prevalence of overall morbidity is high, with 28.3% of the population reporting illness or injury in the previous four weeks. Utilization of the health system is relatively good and 67.1% of these episodes were reported to attend a health provider (predominantly government). The most commonly reported complaint resulting in a health service consultation is fever or malaria – reported in 69.3% of ill children (less than 15 years of age) and 60% of ill adults (15+ years). Non-governmental health providers are also common and work in partnership with government facilities at rural level. Private-for-profit health providers are relatively new and still largely available only in urban areas and large towns. Over-the-counter drugs are increasingly available in rural settings through private pharmacies, shops and kiosks [6]. Nevertheless, the most accessible health service for the rural household, both in socio-economic as well as spatial-temporal terms, is traditional medicine and traditional healers.

### Economic considerations

Coincident with and consequent to having one of the highest malaria burdens, Tanzania is also one of the poorest countries in the world with an annual GDP of $213 USD per capita (2000) and 36% of the population below the basic needs poverty line. Malaria is estimated to consume 3.4% of GDP or about $240 million USD dollars annually [5]. This is stifling for an already fragile economic performance [7]. Tanzania spends about USD $11.37 per person per year on health [8]. Of this, $2.14 is spent on malaria services. About 75% of malaria expenditures are borne by the household, with the government contributing 20% and partners 5% [9]. Of the household malaria expenditure, about one-third is spent on antimalarial drugs and almost half on bed nets, insecticides, coils and other preventive strategies. This burden is greatest on the poorest households and contributes to the continuing cycle of poverty.

### Care-seeking

There have been a number of studies of care-seeking for malaria in Africa reviewed by McCombie in 1996 [10] and updated in 2002 [11] with much additional work since then [12-18]. Many of these studies involve qualitative and sometimes quantitative analyses of data from illness narratives for recalling episodes of recent illness. Common themes emerge which can be summarized as follows: almost every study identified local community or folk perceptions, terminology or explanations of illness that overlap with malaria disease in ways that distinguished fever, malaria and convulsions as distinct in aetiology and required treatment. Care-seeking patterns for simple fever or uncomplicated malaria were more likely managed initially at home while cases with convulsions or severe malaria were more likely to seek care from a health care practitioner. Multiple care-seeking events and switching between types of providers were common. Cases with simple fever or uncomplicated malaria were more likely to seek formal, modern biomedical care and antimalarial drugs, while cases with convulsions were more likely to be managed by traditional healers or traditional practices, as well as modern care. The hierarchy of such events is likely to affect timely access to effective care. One feature of much of this prior work is that, because severe and fatal malaria is relatively rare, nearly all studies based on illness recall ask what people *would do *if they/their child experienced a severe illness such as "degedege" (cerebral malaria with convulsions) rather than what they *did do*.

### Rationale

Although malaria mortality rates are high, fatal malaria is still relatively infrequent when compared to the number of malaria illness episodes. It is possible that the care-seeking patterns of the majority who are ill, but survive, will potentially mask different patterns of those whose care-seeking choices fail and result in a fatal outcome. To understand how best to reduce malaria mortality through improved access to antimalarials, it will be important to examine the care-seeking of individuals who actually died from what they or the health system considered was malaria. No studies in Africa have specifically focused on short-term recall of care-seeking patterns for fatal malaria to see whether and how the general themes above prevail in this sub-group of greatest interest [19]. In this paper an analysis is reported of care-seeking events in a large series of malaria deaths recorded in the course of longitudinal demographic surveillance.

## Methods

### Study area

The general context of malaria and malaria control in Tanzania has been outlined in the background. The specific setting of this study is in the stable perennial malaria transmission belt that runs along the coast of Tanzania and up the Rufiji and Kilombero River basins (Figure 1). This transmission risk is typical of that experienced by the majority (75%) of Tanzanians and of sub-Saharan Africa in general. There are two main rainy seasons, October-December and February-May. The specific data for the study comes from a demographic surveillance system (DSS) in the Rufiji District of Coast Region, managed by the Ministry of Health and the Tanzania Essential Health Interventions Project (TEHIP). Details of the study populations, DSS methods, life tables and results are available for the Rufiji DSS [20]. Household characteristics of the Coast Region are provided in Table 1. These are shown to be generally representative of rural mainland Tanzania.

**Table 1:** General household-level characteristics of Coast Region in comparison to Tanzania rural mainland

**Basic Indicators**	**National Mainland**	Coast Region*
**Household and Housing**		

Average Household Size	4.9	4.9
Percentage of female-headed households	23	18
Percentage of households with a modern roof	43	24
Percentage of households with modern floor	25	10
Percentage of households with modern walls	25	1
Percentage of households with electricity	12	6
Percentage of households using a toilet	93	98
Mean distance to firewood (km)(rural households only)	3.1	1.7
Mean distance to a shop (km)(rural households only)	1.8	1.0
Mean distance to a bank (km)(rural households only)	37.5	31.3

**Education, Health and Water**		

Percentage of adult men without any education	17	24
Percentage of adult women without any education	33	52
Percentage of adults literate	71	58
Primary net enrollment ratio	59	56
Percentage of individuals ill in 4 weeks before survey	28.3	34
Percentage of ill individuals who consulted any health provider	69	83
Percentage of above who consulted a government provider	54	69
Percentage of households within 6 km of primary health facility	75	69
Mean distance to a dispensary / health centre	4.7	3.5
Mean distance to a hospital (km)	25.6	25.9
Percentage of households with a protected water source	57	23
Percentage of households within 1 km of drinking water	55	51
Mean distance to a primary school (km)	1.8	1.7
Mean distance to a secondary school (km)	12.6	13.1

**Economic Activities**		

Percentage of adults whose primary activity is agriculture	63	62
Percentage of children age 5–14 years who are working	62	57
Mean area of land owned by rural households (acres)	6	2.9

**Consumption and Poverty**		

Consumption expenditure per capita (2000/01 TZS / month)	10,120	9,922
Percentage of consumption expenditure on food	65	71
Percentage of population below the food poverty line	19	27
Percentage of population below the basic needs poverty line	36	46

* Rural result provided where available; ** Exchange rate, January 2001: TZS/USD = 803 Source: Government of Tanzania, National Bureau of Statistics, Tanzania Household Budget Survey 2000/01

Rufiji District is 178 km south of Dar es Salaam on the Indian Ocean coast and has a population of 203,000 in 2002 in an area of 14,500 km^2^. The district is entirely rural with 94 registered villages, no urban areas or towns, and has a large area set aside as a game reserve. The economy is predominantly subsistence farming and fishing. Rufiji district is home to several ethnic groups. The largest is the Ndengereko who, according to oral tradition, are the original inhabitants of the area. Other groups include the Matumbi, Nyagatwa (concentrated in the delta area), Ngindo, Pogoro and Makonde. The majority of the people are Moslems (98%) with a few Christians (1.3%) and followers of traditional religions. In addition to local languages, Kiswahili is widely spoken; English is not commonly used in the area. The population has access to 57 formal health facilities: two hospitals (one government and one NGO), five health centres with in-patient facilities (all government) and 50 outpatient dispensaries (46 government). Over-the-counter drugs are available from many private shops and kiosks in the villages. People also obtain services from traditional healers including traditional birth attendants. Immunization coverage ranges from 85% for BCG (tuberculosis) to 66% for measles in children 12–23 months of age. Acute febrile illness and malaria are the leading causes of attendance at health facilities, and the largest cause of mortality. For malaria, the district provides Integrated Management of Childhood Illness (IMCI), Intermittent Presumptive Treatment of malaria in pregnancy (IPT), and first, second and third line antimalarial services at all formal health services, as well as social marketing of insecticide-treated nets (ITNs).

### Demographic Surveillance

The Rufiji District hosts a sentinel DSS area that covers 1,800 km^2 ^north of the Rufiji River and west of the Rufiji Delta (7.470 to 8.030 south latitude and 38.620 to 39.170 east longitude). The Rufiji DSS monitors a total population of 85,000 people in 17,000 households in 32 villages. All residents are registered in the system and all births, deaths, in-migrations, out-migrations, pregnancies and other vital events are monitored and registered. Events are recorded in the Demographic Surveillance Area (DSA) by 150 village key informants and verified by DSS staff. Twenty-eight full-time enumerators update the population register every four months by household survey cycles. The field and data system is based on the Household Registration System Software [21]. The database also includes key household level information on household structure, socio-economics and assets, food-security and environmental features that are updated annually. All households and community structures have been geo-located by global positioning satellite (GPS) systems. The Rufiji DSS is part of the Ministry of Health's National Sentinel System (NSS) for monitoring health and poverty status and serves as a sentinel for rural coastal districts. Annual Burden of Disease profiles are produced by the DSS and used for district planning purposes in the NSS.

### Verbal Autopsy

The Rufiji DSS continuously records vital events within households and among individuals over time in a systematic way. The vital events reporting system consists of key informants who notify the system of any death occurring in the DSS area. This information is passed to a DSS key informant supervisor (or DSS enumerator who informs the key informant supervisor). The key informant supervisor visits the households in which death has been reported within two weeks and contacts the DSS data centre for verification of the registry status. A verbal autopsy (VA) (post mortem interview) is then scheduled and administered to one of the deceased's relatives or the individual who is most well informed of events and details of illness of the deceased. A DSS VA supervisor, who is also a trained clinical officer or health officer, conducts the VA interview. Respondents are not aware of the health care qualifications of VA interviewers. Enumerators also ascertain death events at fixed enumeration rounds three times per year, using specific event forms that are reconciled with the mortality database. There is no population sampling. The entire population of the DSS area is in the DSS and all deaths to DSS residents are subject to VA. Population compliance in both the DSS and VA interviews was very high resulting in high completeness of death registration for registered members. Verbal autopsy was available on 97.7% of deaths, missing only those where the family out-migrated shortly after the death or declined the VA interview.

The VA tool used is that of National Sentinel System [22] based on an evolution of forms developed by the Adult Morbidity and Mortality Project (AMMP) [23] and very similar to that proposed by INDEPTH . It uses individual specific standard questionnaires for: a) children under 31 days of age; b) children under five years but 31 days and older; and c) population aged five years and older. The questionnaires and responses are in Kiswahili. Information such as household ID number, name, age and sex are re-collected for confirmation. In addition, data is collected by open-ended and closed questions on history of events leading to death, together with previously diagnosed medical conditions as well as signs and symptoms before death. Questions about use of health facilities prior to death, reasons for using or not using a particular health facility and confirmatory evidence of medical care and cause of death (if available) are also asked and recorded in the questionnaires. A typical bereavement interview in the course of a VA takes 45 to 60 minutes.

The tentative cause of death is established from the sequence and severity of signs and symptoms, as well as the available confirmatory evidence, by the VA supervisor and recorded on the forms. However, it is physician coding that determines the final cause of death that is subsequently entered in the database. Completed questionnaires are coded independently by two physicians according to a list of causes of death based upon the tenth revision of the International Classification of Diseases. A third physician independently codes the VA in case of discordant results from the first two physicians. Where there are three discordant codes, the cause of death is registered as undetermined (about 6% of cases). A single cause is assigned as the main cause, with contributing causes also indicated. All death coded as the following were included as suspected to be directly or indirectly due to malaria and included in the study: acute febrile illness 1–4 weeks; acute febrile illness < = 7 days; acute febrile illness including malaria; acute febrile illness with convulsions; acute febrile illness with anaemia; cerebral malaria; fever plus malnutrition; malaria; malaria confirmed; and unspecified acute febrile illness.

### Quantitative methods

All data from the DSS and the VA were entered, cleaned and managed using FoxPro (Microsoft Corp). Databases were linked and selected data transferred to Stata 7.0 (Stata Corp) for analysis. The VA database was linked to the household registration database to obtain other indices, such as the socio-economic status. In a separate study, we determined socio-economic indices for individuals in 14,440 rural households in the Rufiji DSS area for the year 2000. The index was based on principal components analysis of the presence or absence of items from a list of 22 specific household assets and nine household characteristics dealing with household ownership, construction features, water supply, sanitation and type of fuel. Further details on the socio-economic index are provided elsewhere [24]. The household index was applied to each individual in the respective household and all deaths due to malaria were partitioned into socio-economic quintiles by this index. Univariate analyses were used to assess the affect of age, sex, socio-economic status, household headship and severity of malaria on initial choices from 13 potential categories of health care providers. Chi-square was used to identify significant factors associated with choice of care sought during the final illness.

### Qualitative methods

The health behaviour research component of the Tanzania Essential Health Interventions Project (TEHIP) investigated the care-seeking and compliance patterns for malaria in a separate study in the Rufiji District from 1998–2001. Eight villages were purposely selected to include four villages with a local health facility and four villages far from a health facility. From these villages 80 households with children under-five years of age were selected by simple random sampling. Ethnographic approaches (semi-structured interviews, case histories and focus group discussions) were used to explore and describe households' responses to childhood illnesses including malaria. A two-step coding strategy was used. In the field, a research assistant, using a provided guide, performed initial thematic coding of the data. Field coding was supervised and consistency checked by a senior social scientist. At computer data entry level, field codes were replaced by corresponding thematic codes written in text macros by experienced data clerks. A data manager supervised the data entry and was responsible for quality and further consistency checks. All qualitative data was processed in a text editor and analysed using text analysis software, Text-Base Beta (Centre for Qualitative Research, University of Aarhus, Denmark). The codes allowed retrieval and compilation of text segments of interest for thematic analysis.

### Terminology

The ethnographic literature on treatment seeking in Africa uses a variety of terms, none of which are wholly satisfactory in capturing the nature and complexity of available health systems. In this paper the term "modern care" is used to describe what conventionally includes biomedical, western, pharmaceutical, professional, official or formal health care and the term "traditional care" is used to describe what conventionally includes traditional medicine, traditional healers, traditional providers, lay providers, traditional practices or folk care.

### Ethical Considerations

All household visits, surveys and questionnaires in the DSS and TEHIP surveys were administered with individual informed consent. All individual and household data are confidential. All reports are based on summary data that cannot be linked to individuals or individual households. The Ministry of Health, National Institute for Medical Research's Tanzania Medical Research Coordinating Committee has approved the research protocols of TEHIP and its Rufiji DSS. Information is fed back to the communities concerned on a semi-annual basis and provided to the local council authorities and the Ministry of Health for planning purposes on an annual basis.

## Results

### Qualitative themes: illness terminology

Qualitative studies confirmed that the population refers to the signs and symptoms associated with the biomedical condition of malaria as three distinct conditions, each with its own aetiology, treatment-seeking patterns and prognosis. The three conditions are: "homa" (fever, vomiting, feeling cold, loss of appetite, limp body, red eyes, not considered life threatening); "malaria" (high fever, vomiting, loss of appetite, feeling cold, some caretakers considered life threatening) and "degedege" (high fever, loss of appetite, stiffness of body, rolling of eyes, lips twisted sideways, twitching, considered life threatening). These are well recognized by most households in the study population.

*" [...] you are able to recognize an episode of degedege in one day. It begins with mild fever and the next day the fever becomes more severe and results in symptoms of epilepsy. The child opens the eyes wide and the black spot cannot be seen, he begins to twist the arm and leg, and then, even if you pour cold water over the child, does not react..." *(Female respondent aged 37 from Bungu – Rufiji).

Although the local population distinguish between the illness "homa" and malaria the distinction is not always very clear to them. Analysis of case studies revealed that the illness term "malaria" has been obtained from modern health care. When mothers take their children to these health services with what had been diagnosed at home as "homa", they are told it is malaria. The following is illustrative of experiences reported:

*"I first thought it was normal homa (fever) and I could see the child had homa. Now, when I took the child to the hospital, they checked the child's blood and informed me the child had malaria. [...] the child was not playing. I touched the child and the body was like fire (mwili wake ulikuwa wa moto), the body was very hot". *(Female respondent aged 29, Bungu, Rufiji).

Anaemia is not often recognized, and where recognized, is not associated with malaria.

### Qualitative themes: aetiology

Although "homa" and especially "malaria" were seen as associated with malaria and mosquitoes, in most cases the signs and symptoms of "degedege" are not attributed to malaria. Life threatening malaria with convulsions is not only perceived as a different illness from malaria through local symptom definition but is attributed to different causes than malaria. Few households mention the mosquito as a cause of the illness described as "degedege". Popular beliefs as to the cause of "degedege" were found to include: fever, evil spirits and a change in weather/wind. The following translation is typical of quotes obtained from respondents on perceived causes of "degedege":

*"....Evil spirits or demons cause degedege. If it happens that evil spirits or demons pass in front of the child, then the child is likely to get degedege. This may result in paralysis of the body or leg or arm or any part of the body..." *(Male respondent aged 46, from Kilimani, Rufiji).

### Qualitative themes: Care-seeking pattern

"Homa" and "malaria" are seen as conditions that can be managed at least initially at home with modern medicine available from shops and from health facilities. But "degedege" is perceived as a serious life-threatening condition for which prompt treatment-seeking is required. People reported different sources of care they used for the treatment of "degedege". These sources encompass more than the biomedical health system and fall into three broad categories: home treatment, traditional healers and biomedical. Home treatment was reported to include the use of modern medicines, such as aspirin from local shops, in the early stages the illness. If the illness reaches a severe stage (convulsions) people claim to use traditional healers in the home or outside the home. Biomedical care ranging across government hospitals, health centres, dispensaries and equivalent private facilities was used in the later stage, when convulsions had subsided. However, some respondents perceive traditional healers as not competent to deal with such illness and claim to seek care from biomedical providers at the beginning of the illness.

*"We use traditional remedies only to treat degedege. They (*remedies*) must have a very bad smell for this will chase away the evil spirit. It is just like telling you to stay in the latrine; surely you will have to find another place because of the bad smell. This is just the same case for the evil spirit attacking the child because of the bad smell it will have to find another place to stay...." *(Male respondent aged 57 years, Kiomboni, Rufiji).

*"I had gone to the dispensary for treatment; my child was suffering from homa. The first day he was given panadol tablets and chloroquine injection and was asked to return the next day for chloroquine injection. The next day while I was there at the dispensary waiting for treatment my child started convulsing. This I believed to be a sign of degedege. Immediately I left the dispensary in search of a traditional healer. Degedege is never treated in the dispensary. Child may die after being injected." *(Female respondent, 39 years old, Kiomboni, Rufiji).

*"When my child developed degedege I was at Kibiti. I had to look for transport to take the child to Songa Hospital (Mchukwi Missionary hospital). There you have reliable service because you find almost all kinds of investigations. I don't like going to traditional healers because they are not reliable and do not have equipment to investigate well your child. They end up telling you things related to superstition." *(Female respondent aged 42, Bungu, Rufiji).

### Quantitative results: care-seeking pattern

In the period January 1999 to December 2001 inclusive, the Rufiji DSS conducted 243,042 person years of follow-up. In this series, 3,023 deaths occurred to resident members and 2,953 (97.7%) verbal autopsies were conducted. Of these, 24.4% (722) had a cause of death suggestive of malaria as the direct or underlying cause, of which 44.3% (320) were in children less than five years of age. Among these child deaths, there was no difference in frequency between sexes, with 51.3% being male and 48.7% female. Of the child malaria-attributed deaths, 282 (88.1%) sought care at least once before death, while 38 (11.9%) did not, or could not, seek care. Convulsions (possible cerebral malaria) were recorded in 30 (9.4%) of these fatal cases.

**Figure 2:**
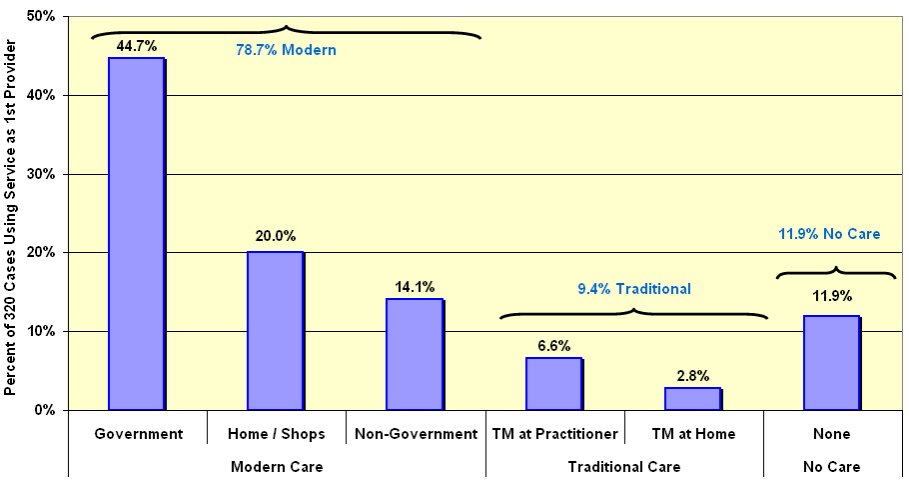
**Initial care-seeking patterns. **Care of first resort sought during the final illness by 320 fatal "malaria" cases in children less than five years of age in the Rufiji DSS sentinel area, 1999–2001.

The verbal autopsies contained both an open-ended narrative account of the final illness and a specific chronological account of where and in what sequence care was sought. There were 13 possible sources of treatment that were collapsed for purposes of certain analyses into three sub-categories of care types (Modern Care; Traditional Care; and No Care) and into six sub-categories of provider types (Government; Home/Shops; Non-Government; Traditional Medicine at Home; Traditional Medicine at Practitioner; and No Care). Table 2 compares the level and detailed source of initial care in acute febrile illness (malaria) for children less than five years of age compared with older cases. The initial treatment-seeking choice for children less than five years of age was modern care (78.7%), whereas only 9.4% used traditional care initially. The remainder (11.9%) sought no care (Figure 2).

**Table 2:** Level and source of initial care in fatal acute febrile illness / malaria by age group in the Rufiji DSS sentinel area, 1999–2001

Level of Care	Provider	Age	
		<5	5+
Government	VHW	0.0%	0.7%
	Dispensary	19.4%	11.2%**
	Health Centre	20.0%	14.4%*
	Hospital	5.3%	5.0%
Home	Mothers	2.5%	2.2%
	Family	9.4%	13.2%
	Drug Shops	8.1%	20.6%**
Non-Government	Dispensary	10.3%	5.5%*
	Health Centre	1.6%	2.0%
	Hospital	2.2%	2.5%
	TM at Practitioner	6.6%	6.5%
	TM at Home	2.8%	1.7%
None	None	11.9%	14.3%
		100%	48%
	Number	320	402
	Total		722

* Significant at 5% level; ** Significant at 1% level TM Traditional Medicine or Practice

Within modern care, government providers were most prominent (44.7%) followed by home care with antimalarials from private shops (20%) (Table 3). Children were statistically more likely to be taken to government health centres and government and non-government dispensaries and less likely to be served by drug shops as the initial resort to care (p < 0.05). There were no significant differences between treatment-seeking patterns for male and female patients regarding the broad choices of modern, traditional or no care. Even though there was no difference in the proportion of males and females receiving traditional care, within the traditional care group, females were statistically more likely to be kept home to receive traditional medicine, and males were more likely to be taken out of the home to see a traditional healer (p < 0.05). There were no significant differences in specific or general care-seeking patterns by sex of the household head. There was no difference in treatment-seeking patterns when comparing choices made by households in the poorest quintile and households in the least poor quintile.

**Table 3:** Type and provider of initial care in fatal acute febrile Illness / malaria by age group, sex, socio-economic status, and type of illness in the Rufiji DSS sentinel area, 1999–2001

Type of Care	Provider	Age		Sex of Child		Sex of HH Head		Poverty Quintiles		Convulsions	
		
		<5	5+	Male	Female	Male	Female	Poorest	Least Poor	With	Without
Modern Care	Government	44.7%	31.1%**	46.4%	42.9%	38.6%	33.3%	42.6%	51.0%	55.6%	43.3%
	Home / Shops	20.0%	36.1%**	21.3%	18.6%	28.2%	31.9%	22.2%	15.7%	19.4%	20.1%
	Non-Government	14.1%	10.0%	12.2%	16.0%	12.6%	9.8%	9.3%	9.8%	2.8%	15.5%
Traditional Care	TM at Practitioner	6.6%	6.5%	7.3%	5.8%	2.1%	2.9%	7.4%	5.9%	16.7%	5.3%*
	TM at Home	2.8%	1.7%	1.2%	4.5%	6.0%	7.8%	1.9%	3.9%	0.0%	3.2%
No Care	None	11.9%	14.4%	11.6%	12.2%	12.0%	14.3%	16.7%	13.7%	5.6%	12.7%
		100%	33%	100.0%	100.0%	100%	100.0%	100%	100%	100%	95%
	Number	320	402	164	156	485	204	54	51	36	284
	Total		722		320		689		105		320

* Significant at 5% level; ** Significant at 1% level TM Traditional Medicine or Practice HH Household. Note, 33 households had a change in headship during the study period and were excluded from the analysis in the sex of HH Head column.

Cases with convulsions were as likely to receive initial modern care as cases without convulsions (77.8% and 78.9% respectively) (Table 3). However, cases with convulsions were less likely to receive no care. Therefore, although the predominant choice of care was modern, inclusion of care from traditional healers was significantly more frequent in those with convulsions than in those without convulsions (p < 0.05). All traditional care was provided by traditional healers and no case claimed to give traditional medicine at home, which is contrary to what is often described in non-fatal treatment seeking.

**Figure 3:**
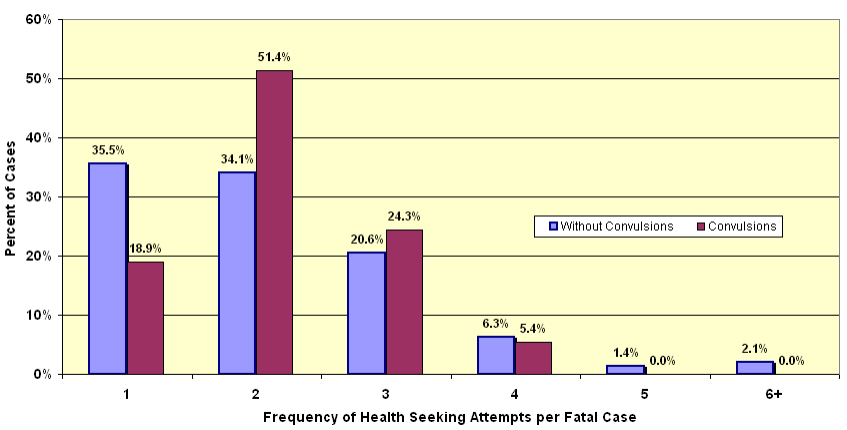
**Frequency of care-seeking events. **Distribution of frequency of care-seeking events at differing categories of provider among those who sought care during the final illness in fatal episodes of malaria in 320 children under five years of age with (dark shading) and without convulsions (light shading).

**Figure 4:**
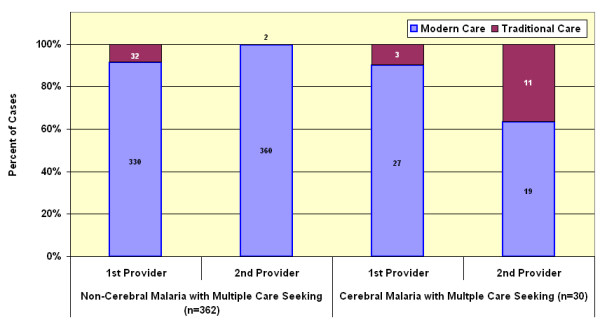
**Loyalty to first provider. **Comparison of loyalty to first provider of modern or traditional care during the final illness in fatal cases (all ages) that saw two or more providers.

Among children for whom care was actively sought, 82.4% of those with convulsions and 90.3% without convulsions sought modern care as the initial care (Table 3). Multiple episodes of care-seeking were common. More than half of cases had two or more treatment-seeking events for the same illness involving a different type of provider (Figure 3). There is also a difference in pattern when initial care choices and cumulative care choices are compared (Table 4). The latter indicates important switching between providers over time and this phenomenon is most apparent when comparing malaria without convulsions to malaria with convulsions. Multiple provider care-seeking was more common if convulsions were present. These synchronic choices (frequency of use of a particular resort to care) are shown in Figures 3 and 4. In the multiple-care-seeking group, switching between modern care and traditional care can be a factor in the delay of effective care. Of the multiple-care-seeking group that did not have convulsions, 88.4% and 99.4% had used modern care at least once by their first or second choice respectively. In this group, of those who started with modern care, only 0.9% switched to traditional care as the second choice. Of the few who started with traditional care as their first choice, most (94%) switched to modern care for their second choice. For the group that had convulsions, 90% chose modern care as their first choice, but by the second choice, 29.6% switched to traditional as the second provider. Switching did not seem to be based on differences in likelihood of receiving treatment. All provider categories were generally able to supply the expected treatment, the poorest being government providers who were able to give treatment for 94% of cases and the best being traditional healers at 96.8% of cases.

**Table 4:** Level and source of accumulative care in fatal acute febrile illness / malaria, all ages, in the Rufiji DSS sentinel area, 1999–2001

Level of Care	Provider	Cumulative Events	
		
		No.	%
Government	VHW*	5	0.8%
	Dispensary	92	14.5%
	Health Centre	104	16.4%
	Hospital	67	10.6%
Home	Mothers	19	3.0%
	Family	64	10.1%
	Drug Shops	36	5.7%
Non-Government	Dispensary	77	12.2%
	Health Centre	39	6.2%
	Hospital	30	4.7%
	TM** at Practitioner	73	11.5%
	TM** at Home	27	4.3%
	Total care seeking	633	100.0%

VHW* Village Health Worker; TM** Traditional Medicine or Practice

## Discussion

Limitations of verbal autopsy methods for malaria deaths have long been recognized, especially with regards to specificity and sensitivity [25,26]. This has provoked efforts to improve and validate verbal autopsy procedures in the settings in which they are used [23,27-34]. The general consensus is that, although imperfect, verbal autopsies are reasonably reliable in determining major causes of death at population level, but may not be suitable for detecting specific impacts of interventions. However, recent work applying adjustments for sensitivity and specificity at differing prevalence levels based on validation studies shows how VA data could be used to monitor progress towards malaria-specific mortality reduction [35].

It must be emphasized that not all of the cases identified as "malaria" in this series are malaria, especially those with unspecified acute febrile illness at older ages. Undoubtedly, some malaria deaths were coded as a cause other than malaria-related. For example, severe and life threatening anaemia, likely to be due to malaria, is prevalent in young children over six months of age in the study area [36] yet VA coded deaths due to anaemia with malaria are infrequent. Despite improvements in verbal autopsy methods in recent years, any study based on verbal autopsy is subject to bias. The recall abilities of respondents can be faulty, although for major events such as a death in the family, it tends to be better than recall of less significant events [33]. In the current study of care-seeking as reported in verbal autopsy, respondents might inflate the number of care-seeking events or exaggerate the choice of modern care if they perceive the DSS to be an instrument of the modern health system or if they feel guilt regarding the care-seeking decisions they took. This would tend to bias responses in favour of more modern care.

Much has been learnt in recent years concerning treatment seeking for malaria in Africa, largely through ethnographic research on illness recall narratives [10-12,14,37-42]. This literature confirms that, for the majority of cases deemed as uncomplicated malarial fevers, modern care based on antimalarial drugs is favoured over traditional medicine. Usually treatment starts at home using anti-pyretics and antimalarials obtained over-the-counter from local shops or left over from previous episodes. Knowledge of appropriate treatment regimens is lacking on the part of the public as well as on the part of private providers [43,44]. Under-dosing in home-based care is common. Malaria is perceived by adult care givers as a mild disease, and if it becomes serious or life threatening, then, it is generally believed that the perceived diagnosis changes from malaria to something that is more likely to be treated with traditional medicine or practices. These beliefs are not rigid. Every case is subject to a process of continuing debate and re-evaluation such that modern pharmaceuticals are also sought, albeit with delay, when convulsions fail to resolve or reoccur after traditional medicine [16,45].

If this is the case in studies of illness recalls, where most patients recover, the question remains whether this general and widespread pattern of treatment seeking holds in those cases where effective treatment seeking clearly failed and the patient died. Since most cases of malaria death in Africa occur at home rather than in health facilities, facility-based data and studies cannot answer this question. The increasing use of demographic surveillance field sites to monitor health at population level in Africa [46] presents an opportunity to examine large series of verbal autopsy findings. Modern verbal autopsy goes beyond cause of death data to collect additional contextual data on, for example, care-seeking events prior to death.

This study confirms that the general patterns seen in illness recalls for uncomplicated malaria in Africa also apply to what people actually do in episodes of fatal malaria in a holoendemic area of Tanzania. Modern care is the first choice for children in over 78% of all child malaria deaths. Government health facilities and shopkeepers were the main source of modern antimalarial drugs. Traditional care may have caused delay in modern care in only 9.4% of fatal cases. 11.9% had no care of any kind. This general pattern held over broad age, sex and socio-economic status groups. Among children with and without the complication of convulsions for whom care was actively sought, 82.4% and 90.3% respectively sought modern care as the initial care (Table 3). In the case of convulsions, although the majority of initial care-seeking was modern, the use of traditional healers increased while the no-care group decreased accordingly. Among those of all ages who sought care two or more times in the course of fatal malaria, modern care was included in the first two choices in 99.4% of cases excluding convulsions and in 90% of cases with convulsions.

Clearly, the perceived severity and danger signs posed by convulsions provoke polyvalent treatment seeking. Nevertheless, modern care is now more popular than previous reports and qualitative studies suggest. One other study of care-seeking patterns in a large series of verbal autopsy reports from the mid 1980's from Tanzania analysed a similar number of all-cause child deaths from Bagamoyo District, a nearby district in the Coast Region [47]. In that study, malaria deaths were not analysed separately, but government providers were the choice in only 45% of deaths. At that time government providers were often without an adequate drug supply and a preference for traditional healers was cited by 41% of mothers as the reason for not using government providers. At the time of the present study in Rufiji, all government providers had adequate drug supplies under the health reforms and offered the integrated management of childhood illness (IMCI) strategy. This could be a factor in the current popularity of government providers.

A relatively small proportion (21.3%) of malaria-attributable child deaths failed to seek modern care (9.4%) or any care (11.9%). This is considerably better than was seen in the mid-eighties, when 55% of children who died had not utilized any modern care [47]. It is also better than seen for deaths in general in the same area during the same period, when 20% of all-cause deaths had no prior care-seeking events [48]. Part of these non-care groups would include those who had sudden death following apparently mild illness, including severe anaemia.

This study shows that most patients now include modern care early in their treatment seeking patterns for eventually severe and fatal malaria, including malaria with convulsions. So why is malaria still the largest single component in the burden of mortality? With belief systems for malaria treatment seeking now firmly on the side of modern care, there is obviously something still failing in 1) the transaction to obtain this care; 2) the quality of the care and referral once it is reached; and/or 3) patient adherence to treatment once it is obtained. This would suggest that policies, efforts and implementation research aimed at improving early recognition of symptoms and danger signs at home, prompt treatment or treatment seeking, the quality and efficacy of the antimalarial available and compliance with the full course of treatment, are now, more than ever, highly justified. When appropriate care-seeking is as high as it is in Tanzania, continuing malaria deaths should be considered as sentinel events deserving of close scrutiny and audit to identify the best remedial strategies for the health system.

There are promising developments. IMCI has recently been introduced in the study area. It places heavy emphasis on training care-givers on early recognition of danger signs and the need for prompt treatment and on improving quality of assessment and care at primary health facilities [36,49]. Replacement of chloroquine with directly observed treatment with sulfadoxine-pyrimethamine (SP) and its simpler single dosing schedule should result in less under-dosing while the introduction of pre-packaged doses has also been shown to be effective in improving provider and client adherence [50,51]. This study was conducted over the last three years of a policy period that used chloroquine as the first line antimalarial. It will be repeated for a similar time frame over the initial three year period of a new policy that uses SP to see if the care-seeking and care-getting patterns change. A qualitative analysis is also planned for the narrative portion of the verbal autopsy questionnaire to look at categories and sub categories of health care related themes in VA reports. This would focus on reasons for delay in seeking modern care (e.g. tried to treat at home without antimalarials, transport, beliefs, poor recognition of severity, lack of confidence in modern care, no power to decide, insufficient finances); delay in receiving modern care (e.g. outside of working hours, weekends, long queues, satisfaction); ineffective modern care (poor communication, no referral, drugs not available, abusive health worker).

## Conclusions

This preliminary study examined what families of children who died from malaria in a holoendemic setting in Africa actually did in terms of treatment-seeking choices and sequence. It confirms that modern medicine in the form of antimalarial pharmaceuticals from shops or government or non-governmental heath facilities is now the preferred choice in an overwhelming majority of cases (78.7% and 97% as their first or second choice respectively). Traditional medicine could only be implicated in a possible delay of modern care in 9.4% of cases. 11.9% sought no care of any kind. There were no differences in these broad patterns of choice by sex of the child, sex of head of household, socioeconomic status of the household or presence or absence of convulsions. Contrary to what is concluded from much of the historical and qualitative work on this subject, modern care is now the care of first choice, even for those who seek care for children with malaria with convulsions (82.4%), although traditional medicine also played an important role in later choices. But despite high rates of modern care-seeking for all forms of malaria, and despite relatively high attendance and utilization of modern care as seen in Tanzania, malaria mortality remains high. This must, therefore, be due to excessive delay in seeking modern care, and/or poor quality of modern care (providers and/or drugs) once sought, and/or poor patient adherence to treatment regimens once obtained.

Certain policy and practice implications arise: 1) public messages need to focus aggressively on improving early recognition of malaria and severe malaria at home and improving promptness of treatment seeking (within 24 hours of onset of malaria symptoms or immediately in the case of severe malaria); 2) quality of modern care providers and modern care must be improved in all sectors, private, NGO and Government; and 3) patient adherence with modern care at home must be simplified and reinforced.

## List of abbreviations

DSA Demographic Surveillance Area

DSS Demographic Surveillance System

GIS Geographic Information System

HBS Household Budget Survey

HH Household

IMCI Integrated Management of Childhood Illness

ITN Insecticide-treated netting

MARA Mapping Malaria Risk in Africa Collaboration

SP Sulfadoxine-pyrimethamine

TM Traditional Medicine

VA Verbal Autopsy

## Authors' contributions

DD conceived the study, participated in the design, coordination and quantitative analysis and co-wrote the article. CM conceived, conducted and analysed the qualitative studies and co-wrote the article. HM led the analysis of quantitative data. EM managed the surveillance system and participated in design and coordination. AM managed and cleaned the quantitative data. YM managed the field work. CM, HK and GR participated in the coordination and management of the study.
